# Social circumstances and cultural beliefs influence maternal nutrition, breastfeeding and child feeding practices in South Africa

**DOI:** 10.1186/s12937-020-00566-4

**Published:** 2020-05-20

**Authors:** Gamuchirai Chakona

**Affiliations:** Department of Environmental Science, Rhodes University, Grahamstown, 6140 South Africa

**Keywords:** Breastfeeding, Cultural beliefs, Dietary diversity, Indigenous knowledge, Infant and young child feeding (IYCF), Paternal inclusion, Social circumstances

## Abstract

**Background:**

Maternal and child undernutrition remain prevalent in developing countries with 45 and 11% of child deaths linked to poor nutrition and suboptimal breastfeeding, respectively. This also has adverse effects on child growth and development. The study determined maternal dietary diversity, breastfeeding and, infant and young child feeding (IYCF) practices and identified reasons for such behavior in five rural communities in South Africa, in the context of cultural beliefs and social aspects.

**Methods:**

The study used mixed methodology technique. Questionnaires were administered to 84 households, pairing mother/caregiver and a child (0–24 months old) to obtain information on maternal dietary diversity, IYCF and breastfeeding practices. Qualitative data on breastfeeding perceptions, IYCF practices, perceived eating habits for lactating mothers and cultural beliefs related to mothers’ decision on IYCF and breastfeeding practices were obtained through focus group discussions.

**Results:**

Maternal dietary diversity was very low and exclusive breastfeeding for the first 6 months of life was rarely practiced, with young children exposed to poor-quality diets lacking essential nutrients for child growth and development. Social circumstances including lack of income, dependence on food purchasing, young mothers’ feelings regarding breastfeeding and cultural beliefs were the major drivers of mothers’ eating habits, breastfeeding behaviour and IYCF practices. Fathers were left out in breastfeeding and IYCF decision making and young mothers were unwilling to employ indigenous knowledge when preparing food (especially traditional foods) and feeding their children.

**Conclusion:**

The study provides comprehensive information for South African context that can be used as an intervention measure to fight against malnutrition in young children. Finding a balance between mothers’ income, dietary diversity, cultural beliefs, breastfeeding and considering life of lactating mothers so that they won’t feel burdened and isolated when breastfeeding and taking care of their children is crucial. Paternal inclusion in breastfeeding decisions and safeguarding indigenous knowledge on IYCF practices is recommended.

## Background

Maternal and child undernutrition remain prevalent in developing countries with about 45% of both child and maternal deaths primarily due to poor nutrition and 11% of child deaths are due to suboptimal breastfeeding [1]. Maternal undernutrition during pregnancy and breastfeeding periods has adverse effects on child growth and development. This is because during this period, maternal nutrient needs increase and if they are not met, mothers may suffer from wasting which limits their ability to fully satisfy the needs of their infants [2]. Nutritious foods and diverse diets which are of good quality and sufficient quantity, are essential for children to meet their nutrient needs and support growth, especially during the first 1000 days of a child’s life which are critical for optimal child growth, health and development [2]. In low income regions, low quality, monotonous diets based mainly on grains and lacking vegetables, fruits and animal-source foods, dominate the diets of many women and children, leading to both maternal and childhood malnutrition [3, 4]. Furthermore, maternal and caregiver education have also been highlighted to have significant impacts on reducing child malnutrition [5, 6]. Appropriate feeding practices are of fundamental importance for the survival, growth, development, health and nutrition of infants and young children [7].

The World Health Organisation (WHO) [8] recommended that mothers worldwide should exclusively breastfeed infants for the child’s first 6 months of life for optimal growth, development and health. Thus, the promotion of exclusive breastfeeding (EBF) during the first 6 months of life is the most effective child health intervention currently feasible at population levels in low-income countries [7, 9–11]. Thereafter, infants and young children should be given nutritionally and adequate, safe, complementary foods from the age of 6 months to meet the evolving needs of their growing bodies. Mothers should continue breastfeeding up to the age of 2 years or beyond as this has numerous benefits for both infant and maternal health [8–10]. For example, breast milk is important as it helps decrease the risk of infectious and chronic diseases, including diarrhoea and respiratory tract infections, especially during infancy [11–13]. Furthermore, mothers with shorter breastfeeding duration have an increased risk of breast and ovarian cancer, type 2 diabetes and hypertension [14–16].

South Africa is among the five Southern African countries that have made no progress between 1990 and 2008 in reducing mortality amongst children younger than 5 years [17]. Although HIV/AIDS has claimed many lives, undernutrition is one major factor contributing to the under-5-mortality rate [18]. In South Africa, exclusive breastfeeding for the first 6 months of life is rarely practised [11, 19, 20], even before the emergence of the HIV epidemic in the country [21]. Although there are high rates of breastfeeding initiation in the country (75–97%) [22], exclusive breastfeeding rates decline to as little as 12% at 0–3 months of child’s age and continue to drop to at most 8% at 4–6 months [23, 24]. Mothers substitute breastfeeding with formula milk feeding [21], even when the government stopped issuing free infant formula since January 2011 [25]. Approximately 35–50% of lactating mothers discontinue breastfeeding before 3 months postpartum and commonly introduce complementary foods^1^ at an early age, sometimes as early as 6 weeks [23]. Mixed feeding is the most common infant feeding practice in South Africa, and has health impacts on the child’s health due to exposure to the risk of diarrhoea and malnutrition [11, 21, 26]. Thus, suboptimal breastfeeding, inappropriate feeding practices, poor nutritional quality diets at weaning^2^ and early introduction of solid foods, among other complex factors such as inadequate sanitation, are contributing to life-threatening infant and child health problems in the country. Childhood undernutrition in the form of stunted growth and underweight persists while overweight is increasing [27, 28] making these major nutritional disorders. Thus, many households and communities in the country suffer from the double burden of malnutrition from the dual nutritional problems of deficiencies and hunger whilst some may suffer from obesity and related diseases [5, 29].

In this context, increasing breastfeeding rates is a strategic priority in South Africa. However, understanding the determinants of breastfeeding behavior as well as IYCF practices is a critical step towards the prevention of undernutrition in young children and decreasing the risk of infant morbidity and mortality within vulnerable settings in the country. Maternal characteristics such as age and education [5, 6], household demographic such as household size and household head and socioeconomic factors such as income and employment status, have been implicated in driving poor IYCF practices [30]. Furthermore, cultural beliefs such as food taboos and perceptions by mothers, households and communities also influence breastfeeding behavior and complementary feeding, including the types of foods young children are given [31]. Grandmothers’ knowledge and their decisions were also regarded as critical for early child feeding practices in other African countries [32–34] and Nepal [31].

To date no study in South Africa has integrated in a single study, all the above-mentioned factors that affect child growth and development, yet the country is suffering from the double burden of malnutrition with high rates of infant mortality. The present study explored maternal dietary diversity, breastfeeding behavior, IYCF practices and the factors that influence these in rural and peri-urban settlements in South Africa. The research questions were designed to gather information on infant care giving practices including breastfeeding, children’s diets (including preferred and restricted foods), maternal and child dietary diversity and household socio-economic characteristic. Questionnaires were used to obtain breastfeeding information, diets and dietary diversity for mothers, caregivers and children in selected households within the Kat River Valley communities. The socioeconomic characteristics included household size, age of both respondent and child, maternal education level, gender of household head, source of income for mother/caregiver and household head, and food expenditure per month.

Community’s perceptions on breastfeeding (including mother’s behaviour and experiences) and IYCF practices in the context of cultural beliefs, nursing mother’s diets and dietary changes, grandmothers’ knowledge regarding children’s diets and decision-making regarding breastfeeding and IYCF practices were also explored through focus group discussions. Perceived changes in the children’s diet through reflection on the past and present diets, and the drivers and barriers to such dietary changes were noted in the study.

## Methods

### Study area

The study was conducted in five settlements (Hertzog, Balfour, Ekhupumuleni, Blinkwater and Ntilini) along the Kat River Valley in the Eastern Cape province of South Africa. The area is home to about 50,000 people, mainly IsiXhosa (84%) [35], which made it ideal for a study involving cultural beliefs and indigenous knowledge in the African context. The majority of the households are female-headed and many households rely on food purchasing. Education levels are poor with approximately 9.7% of the population having a matric^3^ qualification and none was recorded as having a tertiary qualification [35]. The area is characterized with high unemployment rates [35] with 90% of households receiving either an old age pension, disability grant of R1570 per month or child support grant of R480 per month. The South African Rand to US Dollar exchange rate was approximately 12:1 at the time.

### Sampling

This study was conducted in February and March 2018. A ‘mixed suite’ [36] of research tools, including both quantitative and qualitative techniques, was used. These included household surveys and focus group discussions. All interviews were conducted in the respondent’s preferred language of isiXhosa or English. Both translators and enumerators were trained on how to conduct interviews using the questionnaire so as to provide full understanding of the administered questions. Ethics approval was granted by the Rhodes University Ethical Standards Committee on 6 November 2017 with reference number 8628531. Although seasonal variation is known to have an effect on local diets, nutrition and food access, this does not apply to South African households as many depend on food purchasing [37] using social support grants which they receive on monthly basis. However, sampling was spread throughout the month to cater for the times when households had received their government support grants.

### Quantitative study: household surveys

#### Procedure

The study used a purposive sampling approach which is a nonrandom, key informant selection technique to gather data using the deliberate choice of an informant based on the qualities that the informant possesses [38]. Here, we targeted households with mothers or caregivers with infants and/ or young children aged 0–24 months. Mothers or caregivers (ideally the person in the household who takes care of the child) were interviewed to gather information on their individual diets consumed in the previous 24 h using the standard 24 h recall technique [39, 40]. Eighty-four pairs of child-mother or child-caregiver were available for interviews across the communities. Of these pairs, 48 were mother-child whilst 36 were caregiver-child pairs which included 21 grandmothers and the other 15 pairs were other female family members including aunties. Individual dietary diversity scores and the nutrient adequacy in the women’s diets were measured following FAO and FHI 360 [40] and Martin-Prével et al. [41]’s minimum dietary diversity for women (MDD-W) of reproductive age (WRA) indicator. The MDD-W indicator is the sum of food groups consumed by women over a reference period, and it can also be used as a measure of household access to a micronutrient rich diet [42, 43]. Therefore, MDD-W is noted as a conservative estimate of household nutritional security as well as micronutrient adequacy of the women’s diet [40, 41].

The women were asked to recall and name all the food they had consumed in the past 24 h (day and night), that is, all dishes, snacks, and drinks. They were encouraged to remember all the food consumed per meal and in-between meals, fully describing all the ingredients in mixed dishes. For example, if they had rice and beef stew for lunch, they were supposed to list all the ingredients that were used to make rice (e.g. rice, water, salt) and beef stew (e.g. beef, water, cooking oil, salt, carrots, onion, tomatoes, potatoes, spices etc.). All the ingredients were then coded by the researcher into a list of 14 major food groups which were aggregated to ten for analysis [40, 41]. Following FAO and FHI 360 [40], MDD-W was calculated as the sum of food groups consumed by a woman from the total of ten specific food groups required, which consider nutrient-rich foods including animal-source foods; fruits and vegetables; and pulses, nuts, and seeds. The ten food groups included: (1) Grains, white roots and tubers, and plantains (also known as starchy staples); (2) Pulses (beans, peas, lentils); (3) Nuts and seeds; (4) Dairy; (5) Meat, poultry and fish; (6) Eggs; (7) Dark green leafy vegetables; (8) Other Vitamin A-rich fruits and vegetables; (9) Other fruits and (10) Other vegetables. These food groups included in the MDD-W mostly reflect the diet quality with the probability of minimum micronutrient adequacy of the women’s diets summarized across 11 important micronutrients which are vitamin A, thiamine, riboflavin, niacin, vitamin B6, folate, vitamin B12, vitamin C, calcium, iron, and zinc [41]. The Fats and oils food group was not included for MDD-W as this do not contribute to the micronutrient density of the diet [40]. Using MDD-W allows grouping women into classes of food secure or food insecure, therefore, women were grouped into these classes. A woman was considered as having poor dietary diversity and food insecure if she had consumed < 5 food groups or had achieved MDD-W with good dietary diversity and was food secure if she had consumed ≥5 food groups in the previous 24 h [40].

Surveys also included open-ended questions on breastfeeding and IYCF practices, specifically on the perceived “proper” diet for children and what mothers or caregivers actually fed their children with, if there were any foods which they avoided or did not give to children, as well as if mothers consumed different foods to their children and the reasons for this. Food group intake for infant and young children up to 24 months of age was determined using the seven-category child indicator as recommended by the World Health Organisation (WHO) [44]. The child indicator only represents the complementary foods in the diet, excluding breast milk intake, where children are expected to consume a minimum of ≥4 food groups per day out of the seven recommended. The seven recommended food groups for children are: (1) Grains, roots and tubers, (2) Legumes, nuts and seeds, (3) Dairy products, (4) Flesh foods (meat, fish, poultry and liver/organ meats), (5) Eggs, (6) Vitamin-A rich fruits and vegetables and (7) Other fruits and vegetables.

#### Analysis and presentation of results

Data were entered and cleaned using Microsoft Excel and quantitative data were analysed using Statistica version 12 (StatSoft Inc., Tulsa, OK, USA). Descriptive data are presented as means and standard deviations (SDs) (mean ± SD) and percentages and are presented as tables.

### Qualitative study: focus group discussions

The study also implemented focus group discussions (FGDs) which actively make use of group interaction on the issues relevant to a specific topic. These gave insights into collective meanings attached to breastfeeding behaviour, IYCF practices and maternal food consumption behaviour that could not be elicited through questionnaires as participants were able to build on each other’s ideas and comments. For example, we were able to obtain information on the cultural beliefs and feelings of individuals and why they acted in the way they did. Like other qualitative research methods, these FGDs were used to develop an understanding of the meaning and experiences of peoples’ lives from the point of view of those who experience it [45, 46].

#### Procedure

The participants who had not participated in the household surveys, were purposively recruited by the community leaders, village or ward leaders who also helped with organizing the venues and times for the FGDs. Information about the study and its purpose was shared with those who were approached and they had the right to agree or decline to participate in the study. Informed consent was obtained from each FGD participant after the researcher had explained the purpose of the study. Nine focus groups were conducted with 7–12 women of mixed age groups particularly young and old women with 94 participants in total (Table 1). The majority of participants had informal employment as those who were formally employed were not available at the times when FGDs were conducted. Participants were informed that they were allowed to withdraw from participation at any time without any penalties and no incentives were offered for taking part in the study. For each FGD, there was an interpreter, translator and an assistant and it took between 60 and 90 min. The principal researcher led all the discussions and both the principal researcher and an assistant made notes of all the discussions and the responses that appeared most often in the group discussions. Group discussions were also recorded, only after consent was provided by the participants.

**Table 1:** Number and age distribution of focus group discussions participants in the Kat River Valley communities

Community	N			Number per age group in years			
	Total	Mothers	Grandmothers	< 20	20–30	31–50	> 50
Hertzog 1	12	6	6	2	3	3	4
Hertzog 2	12	5	7	0	4	3	5
Balfour 1	11	5	6	1	2	4	4
Balfour 2	10	4	6	0	3	4	3
Ekhuphumuleni 1	9	4	5	0	3	1	5
Ekhuphumuleni 2	7	4	3	0	2	2	3
Blinkwater 1	12	4	8	1	2	4	5
Blinkwater 2	10	6	4	2	2	2	4
Ntilini	11	5	6	0	3	3	5
Total	94	43	51	6	24	26	38

The focus group questions did not directly ask about breastfeeding because the researcher wanted to determine if breastfeeding was deemed the best feeding practice for infants and young children by the participants in the study communities. All questions asked in the FGDs were open-ended, formulated from some of the household survey questions, with new questions arising from the responses given in the FGD. The FGDs sought responses to the following core questions:

1. What foods should a lactating mother eat or avoid and is this practiced by mothers in your community?
2. What foods or drinks should be avoided or given to infants or young children who are 0–24 months of age? Why?
3. Are there any changes in the foods that were given to children in the past and now? If so, what is causing the changes?
4. What are the consequences of these changes for the health of the children?
5. What need to be done to promote child health in your community?

#### Analysis

Data from all the FGDs, which were mostly handwritten field notes, were entered into Microsoft Word 13 and were edited. Everything was translated from IsiXhosa to English. Some important IsiXhosa words were written in IsiXhosa with their English meaning given in brackets. The researcher read the transcripts several times to understand the information. During data analysis, we used qualitative content analysis (QCA) which is useful to interpret textual data content by using a systematic classification process that involves coding to identify patterns or themes. This involves four steps: repeated review of the transcript to gain thorough sense of the overall content in the texts, identifying central meaningful units in the material, condensation of the content through a coding of the text, and finally creating categories that contain the condensed meaning of the main themes in the material [47]. Themes were then analysed through coding [48] using NVivo software for qualitative data analysis. Similarities between the codes were identified and those that were connected were combined to form primary themes for the discussions. Most sections of the discussions were quoted verbatim with a few modifications to increase readability.

## Results

### Quantitative study

#### Sample description

The sample consisted of 84 pairs of mother/caregiver and child with mean age of 34.7 ± 11.7 years for mothers/caregivers and 16.3 ± 6.1 months for children. Approximately 56% of the children were female and overall, 21% of the children were never breastfed (for reasons other than HIV/AIDS) (Table 2). Although the initiation of breastfeeding was immediate and prevalent, with 79% of children fed with colostrum, exclusive breastfeeding for the first 6 months of life was rarely practiced; only 36% of the children were exclusively breastfed for the first 6 months of life, with substitute breastfeeding (49%) being common although mixed feeding was practised. Mean household size for the full sample was 6.9 ± 3.6 persons with 56% of the households being female-headed (Table 2). The majority of women had not studied up to matric level, only 29% had matric and 1% of the women had post-matric qualification. About 70% of women received some cash income in the form of social grants (mostly child support of R410 per month) whilst 26% of women did not have any form of cash income. In addition, about 44% of household heads received social grants and 31% were either part-time or full-time employees. Mean food expenditure per month for the households was R1124 ± R667.

**Table 2:** Sample distribution by demographic factors

	Indicator		% (*N* = 84)
Maternal characteristics	Mother/caregiver mean age (yrs)	34.7 ± 11.7	
	Maternal education	Primary (up to grade 7)	20
		Secondary (grade 8–11)	50
		Matric (grade 12)	29
		Post matric	1
	Maternal source of income	None	26
		Child support grant only	61
		Child support grant and other sources	4
		Government grant	5
		Pension	1
		Domestic worker	2
		Trainee	1
Household characteristics	Household head	Female-headed households	56
		Male-headed households	44
	Household head source of income	None	21
		Old age grant	17
		Government grant	27
		Pension	4
		Part-time/full time job	31
	Household mean food expenditure per month	R1124 ± R667	
	Mean household size	6.9 ± 3.6 persons	
Child characteristics	Mean age in months	16.3 ± 6.1	
	Sex of child	Female	56
		Male	44
	Infant breastfeeding practices	Immediate breastfeeding initiation	79
		Feeding colostrum	79
		Never breastfed	21
	Exclusive breastfeeding for first 6 months of life	Mothers continued breastfeeding till: 12.3 ± 6.1 months	36
	Mixed feeding	Mean initiation started at: 2.3 ± 1.4 months	16
	Substitute breastfeeding feeding^a^	Mean initiation age: 2.0 ± 1.5 months	49

^a^This also includes those children who have never been breastfed

#### Maternal dietary diversity

**Fig. 1:**
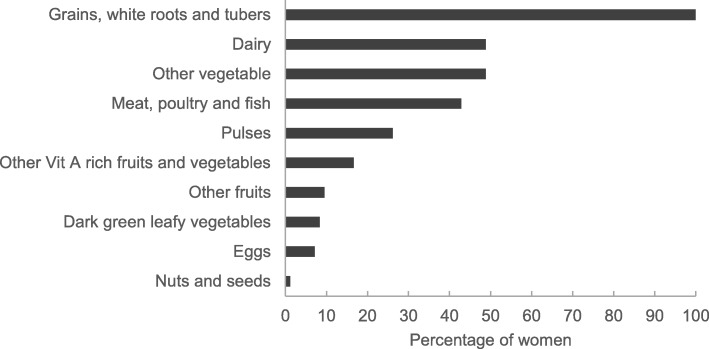
Percentage of women consuming different food groups in the previous 24 h in the Kat River Valley

Dietary diversity scores were very low for women in this study, with a mean of 3.1 ± 1.4 food groups consumed in the past 24 h out of the ten expected. Only 14.3% of the women had consumed at least five food groups implying a good quality diet and being food secure. The majority of the women failed to achieve the minimum dietary diversity recommended for women and were food insecure as their diets lacked essential nutrients. Maternal diets were based mostly on starchy staples with all women consuming foods in the Grains, white roots and tubers group (Fig. 1). All the other food groups were consumed by less than 50% of women, although Dairy products group (mostly sour milk) and Other vegetables group (mostly cabbage and onions) were consumed by 49% of women. Items in the Meat, poultry and fish group were also consumed by a considerable percentage of women followed by Pulses which were consumed mostly as umngqusho (samp^4^ and beans), which is a favourite IsiXhosa dish and a good alternate protein source. Consumption of other food groups such as Eggs, Vit A rich fruits and vegetables, Dark green leafy vegetables, Other fruits and Nuts and seeds was infrequent, only consumed by less than 20% women in the previous 24 h.

#### Children’s diets

As most children under the age of 24 months in this study were not breastfed, mothers/caregivers reported the foods that they considered as good for their children’s growth and development. The foods that were perceived by the majority of these women were mostly starchy staples. The majority of women considered porridge to be the most important meal for children, with 56% mentioning mealie meal porridge, 23% reporting burned flour porridge and 17% mentioned instant mealie meal porridge (Table 3). About 23% of women mentioned mashed potatoes with margarine and 19% perceived baby cereals such as cerelac and nestum to be good for children. All types of vegetables, fruits and dairy products were perceived to be important for child growth and development by more than 30% women. The importance of breastmilk was mentioned by only 19% of women, and meat and eggs were rarely mentioned. Almost all the women perceived that the South African popular snack (niknaks) is unhealthy for children and should not be given to children regularly.

**Table 3:** Foods cited by at least 10% of mothers and caregivers that they perceived should be given to children for growth and development

Food	Food group for dietary diversity	Percentage of women (*N* = 84)
Mealie meal porridge	Grains, roots and tubers^a^	56
Fresh milk and nespray powdered milk	Dairy	39
Vegetables (*cabbage, spinach, mixed vegetables, broccoli, cauliflower*)	All vegetables and fruits group^b^	35
Fruits (*banana, apple, peaches*)	All vegetables and fruits groups	32
Burned flour porridge	Grains, roots and tubers	23
Mashed potatoes with margarine	Grains, roots and tubers	23
Bottled milk (*nan, infacare, nan pelargon*)	Dairy products	19
Nestum and cerelac	Grains, roots and tubers	19
Breast milk	All nutrients, both macro- and micronutrients	18
Butternut and pumpkin	Vit A rich fruits and vegetables	18
Instant porridge	Grains, roots and tubers	17
Carrots	Vit A rich fruits and vegetables	17
Bread with sour milk	Grains, roots and tubers + Dairy products	12
Meat (*chicken livers, lamb*)	Flesh foods and organ meat	12
Oats	Grains, roots and tubers	10
Umphokoqo (*Xhosa traditional dish*)^c^	Grains, roots and tubers + Dairy products	10
Yoghurt	Dairy products	10
Niknaks^d^	Grains, roots and tubers	1

^a^Includes infant cereals

^b^Falls in both Vit A rich fruits and vegetables, and Other fruits and vegetables groups

^c^Dish made from mealie meal and water and served with sour milk known locally as amasi

^d^South African snack, originally cheese puffs which is primarily made from maize

The foods that the mothers/caregivers feed their children with were almost similar to that which they perceived, except for niknaks snack, which most women gave regularly to their children. Porridge was the most common dish provided to children, reported by 57% women. Of these women, 54% feed their children with porridge everyday whilst about 13% of children were eating porridge 5–6 times a week (Table 4). Surprisingly, niknaks snacks, which was perceived as unhealthy for children, was the second most common food which 49% of women gave to their children, with about 64% children consuming niknaks at least five times a week. Children also consumed potatoes frequently as reported by 44% women, with 84% consuming potatoes for at least three times a week (Table 4). All the above-mentioned foods consumed by children falls in the Grains, white roots and tubers group. Dairy products were frequently consumed by children as fresh or sour milk (20%), either with a traditional dish umphokoqo (20%) or with brown bread (13%). The other food groups were rarely consumed with children. For example, vegetables were only consumed by 17% of the children, and of these, 43% rarely consumed them and 36% consumed vegetables sometimes. Similarly, only 11% women reported the consumption of fruits by children, whereby 78% children did so at most four times a week. Eggs, Meat, and Legumes and nuts were infrequently eaten and were mentioned by less than 5% of women.

**Table 4:** Most cited foods that mothers or caregivers feed their children. All values in the table are expressed as percentage

Specific foods given to children	Percentage of mothers (*N* = 84)	Number of times given per week			
		Everyday	Often (5–6 times)	Sometimes (3–4 times)	Rarely (0–2 times)
Porridge (*burned flour and mealie meal*)	57	54	13	25	8
Niknaks snacks	49	15	51	32	2
Potatoes	44	30	22	32	16
Carrots	24	25	5	45	25
Umphokoqo/african salad (*mealie meal and sour milk*)	20	18	24	47	12
Milk (*fresh or sour*)	20	41	18	41	6
Butternut	17	29	0	50	21
Vegetables (*mostly spinach and cabbage*)	17	21	0	36	43
Purity (commercial baby food)	13	18	9	64	9
Brown bread (*mostly served with sour milk*)	13	36	0	45	9
Fruits (*banana and apple*)	11	22	0	78	0
Noodles	11	33	11	33	22
Rice	10	25	0	63	13

Some of the reasons why mothers/caregivers used these diets, especially when feeding their children, are in Table 5. Mothers/caregivers believed that certain foods, especially porridge, were good and healthy foods for their children, and would make the child grow strong and big with lots of energy. The majority of mothers were feeding their children with different foods for health reasons and child’s preferences. However, circumstances such as the food being readily available and cheap, or for no particular reason were also mentioned (Table 5).

**Table 5:** Most cited reasons why mothers/caregivers feed their children specific foods

Specific foods given to children	Reasons for feeding children with the food type	Percentage of mothers/caregivers
Porridge	good for babies	23
	helps child to grow well and become big and strong	29
	for baby’s health, keeps baby strong, gives energy and strength	31
	just giving, its breakfast, easy to prepare and its cheap	10
Niknaks snacks	child enjoys or likes the food	66
	calming the baby and is readily available	31
Potatoes	good for children and they like the food	51
	for the child to get energy, be strong and grow well	22
	its healthy food for children	22
Carrots	good for growth, health, strength and eyesight of a child	85
	just giving when available	10
Umphokoqo	child enjoys it	35
	for the child to grow and become big	18
	to gain energy, strengthen the body and become strong	35
Milk	healthy food for growth	59
	good for the baby	24
	gives child energy and strength	18
Butternut	just giving because it is good for babies	21
	for the growth of the child and it gives energy	50
	healthy food	21
Vegetables	good for health and growth of children	83
	just giving if available	7
Purity	for growth and makes child strong	36
	its healthy	27
	good for babies	36
Brown bread	good for the baby	18
	that is the food readily available and children likes it	36
	for energy and strength of the body	45
Fruits	fruits are healthy and are good for growth of baby	100
Noodles	good for the baby because it is soft and easy to eat	33
	healthy and growth of baby	55
	child loves the food	11
Rice	child enjoys the food	25
	just giving and for the baby to eat	26
	good for growth and the child will be strong	50

### Qualitative study

The analysis for FGDs identified four major themes related to breastfeeding and IYCF practices and these formed contrasting views between young mothers and grandmothers who participated in the discussions. The different themes are discussed below.

#### Exclusive breastfeeding as the best feeding practice of infants and young children

Although the FGDs questions did not directly ask participants about breastfeeding, all participants mentioned exclusive breastfeeding in the first 6 months of a child’s life as the best feeding practice. They reported that breastfeeding keeps the child healthy and after 6 months a child should still be breastfed but may be given other foods with all the necessary nutrients for growth and development. Thus, all participants agreed on the importance of breastfeeding and that it improves their young children’s health which they all perceived should be practised. They also agreed that a mother who is breastfeeding can quickly notice if the child is sick unlike when one is not breastfeeding. One mother said, “*Breastfeeding is crucial because breast milk gives all the nutrients to the baby such that the baby is not affected or get ill even if they refuse to eat other foods*” whilst a grandmother also said, “*Breastfeeding is very important. It also helps you to quickly notice if the child is unwell. The child grows up healthy and not ill every time …*”

However, although they all agreed on the importance of breastfeeding, not all agreed on practicing exclusive breastfeeding for the first 6 months. Young mothers said they resort to supplementing or substitute breastfeeding with bottled milk and other foods which are perceived good for children and they do not see the difference in their children’s health. Mothers felt that sometimes breast milk alone would not be enough for the child as the child keeps crying. They also reported that it’s a better way of feeding a child, especially during times when the mother is unavailable to breastfeed. “*Breastfeeding is good for babies but I was producing less milk and the baby kept on crying. With cerelac and nestum, the baby would eat and be filled. Peragon is also good for children when the mother is not around to breastfeed. Purity especially the butternut and sweet potatoes one, custard and milk help the baby to grow strong and healthy. Water is important to add to breast milk so that the child does not dehydrate”*, said one of the mothers.

#### Social circumstances, cultural beliefs and mothers’ behavior regarding breastfeeding and IYCF practices

The societal pressure for the fear of losing their partners during breastfeeding periods also came out in the FGDs as young mothers mentioned that culturally, they are supposed to practice abstinence from sexual intercourse with their partners for at least 3 months when they are breastfeeding. If not practiced, the breast milk becomes “impure” and may make the child sick. In avoiding this, some mothers may wean their babies at an early stage so as to engage their partners and at the same time not compromising the child’s health. However, grandmothers felt that by not following these traditions, many infants and young children are getting sick as some mothers wean their children when they are too young; others continue to breastfeed children with “impure” breast milk and many lactating mothers give more time to their partners than their babies. A grandmother said, “*Culturally, a mother who have delivered should not be with the husband for up to three months as this would make the milk impure therefore the child can develop a big head, big stomach and becomes very weak. Young mothers now are always with their husbands as soon as they can and the problems persists with the child. They keep disobeying the culture and this is affecting the child to grow well. Most are now giving the babies some other foods at the earliest age.*” Also, a young mother concurred by saying, “*In IsiXhosa culture, soon after delivery a mother should not be with the husband for up to three months so that she concentrates on feeding the baby as the father may make the milk impure. This helps the child to feed well, be healthy and will have a beautiful skin. But because of what is happening in the world, the pressures, I had the fear of losing my husband to other women. Therefore, most young mothers now prefer not to breastfeed their children and satisfy the needs of their husbands to keep them around.*”

However, grandmothers felt bottled milk and other foods given to young children were not good for their health and noted that as the reason why young children in their communities were always sick. “*Mothers these days no longer want to practice breastfeeding which is good for the baby. They give their babies bottled milk which is not good or healthy for the child. It makes the child weak*”, a grandmother said. Grandmothers also perceived that early introduction of solid foods and bottled milk was affecting young children’s health but mothers were not willing to practice breastfeeding for various reasons. A grandmother pointed out that, “*Mothers do not want to breastfeed their babies and they end up giving them bottled milk. When preparing bottled milk, they are not following instructions. In the past, we used to breastfeed our babies up until six months without giving them any other food and continue to as much as 2 years*” whilst another said, “*Early introduction of other foods before the child is even three months is practiced by the young mothers and it is not good and healthy for the babies because their intestines are not ready for the food they are given.*” It was also perceived in the focus group discussions that poor feeding practices for infants and young children and poor eating habits by young mothers were currently compromising children’s health. “*… mothers are not eating good food and breast milk is becoming not good for the baby and is affecting growth and development of the child*”, said one grandmother. They perceived that, due to consumption of poor diets by the mothers, even if they were to breastfeed their children, the breast milk produced was not nutritious enough to provide essential nutrients to their children.

#### Affordability and the cost of food influencing IYCF practices

Young mothers from all focus groups felt that, the choice of food which they consumed and gave to their children was mostly governed by affordability, household food availability and their income rather than their food preferences. A mother said, “*… because money is not always available and we do not work, we all eat what is available in the house. Chips (niknaks) are cheap to buy and children love them, so we give them if they do not want to eat other foods.*” Although their children preferred consuming snacks and juice rather than nutritious foods, they would have been feeding them with good quality foods if they could afford to purchase diverse foods. Participants from all the focus groups agreed that vegetables and fruits were good for their children’s health, but they all noted that it was impossible for them to always have these foods because fruits and vegetables are very expensive to buy. “*… mealie meal porridge with milk, salt, sugar and peanut butter if it is there is excellent for feeding babies. Butternut or pumpkin for good health, carrots mixed with potatoes promote good health for the children. Also, umpokoqo with amasi helps the child to grow big. All types of vegetables and fruits are good but we do not have the money to buy always*”, said a young mother. Most of the time mothers lack money to purchase vegetables and fruits and hence always relied on cheap basics and niknaks which their children liked so much. However, some grandmothers felt it was more of poor caring practices exhibited by the mothers rather than the affordability issue as one mentioned that, “*They (mothers) do not care for their babies’ health as many are not breastfeeding their babies. They give them juice which is sweet and they say babies like it. They do not cook food for their children and buy food which is not healthy like chips (niknaks) and make that a meal. Children should be given home-made meals which is fresh.*”

#### Dietary changes and mothers’ fear regarding preparing traditional foods

When answering the questions on the changes in the foods that were given to children in the past and currently, all participants agreed that there has been a dietary transition regarding children’s diet. However, there were mixed responses on whether these changes had negative consequences on the health of the children or not. Grandmothers perceived that young mothers were not prioritising their children’s diets as they were mostly feeding them with modern foods and mostly not home-made food. They perceived that feeding young children with traditional foods was better for their health even if they were given before 6 months of age because these have more nutrients than modern day foods. However, all participants agreed to this, although young mothers felt it was difficult for them to prepare the traditional foods, and they preferred it if grandmothers prepared the foods because the food can make a child sick if not prepared well. “*We know that traditional foods help babies and young children to have appetite and grow well with a strong immune system and babies do not suffer from different diseases like ring worms, diarrhoea and rash. But these are difficult to prepare.*” said a young mother. Another grandmother also supported this saying “*… inembe (starch water from soaked samp) is given to babies because it helps children to gain weight. Umhoqo (roasted/burned flour porridge) is an excellent food for the babies. It is health and makes them grow big and gain weight. However, if umhoqo is not prepared properly, it affects the child’s development as the child may suffer from diarrhoea.*” Therefore, young mothers felt it was much easier for them to give their children ready-to-eat foods like yoghurt and instant porridge as it is also cheaper to buy. However, grandmothers felt that it was just an excuse by young mothers because they did not want to learn and also did not want to cook food for their young children. A grandmother said, “*In the olden days we used to cook and give babies grinded fresh maize and milk for the baby to grow well and be healthy. Mealie meal porridge and milk is good for children. Nowadays, babies are not breastfed properly, they are fed with nestum and yoghurt which are not good for their health. This is because young mothers do not want to breastfeed their children and they use poor food for feeding because they do not want to cook or learn to cook, they are lazy. As a result, children are growing unhealthy*” whilst the other also said, “*Children these days are given poor diet, too much of instant porridge, purity and yoghurt. They are not given umhoqo (burned flour porridge). Mothers do not want to cook mealie meal porridge which makes children grow strong but give them instant foods. Butternut helps in body building and the baby has a healthy body and good weight. Modern mothers are lazy to cook. They are relying of meals of nestum, purity, instant foods, sweetened yoghurt which are affecting child growth.*” However, some medicinal plants deemed by the communities as good for the health of young children were also mentioned in the FGDs, although these were not directly aligned to food consumption. “*Isicakathi (Salvia scabra L. f.) which is an herbal tea is given to babies and this helps with wind and phlegm which comes out when a baby cough. Therefore, this medicinal plant helps to clean the chest of the new born so that they won’t suffer from these diseases. The plant also cleans the baby so that they do not suffer from rash or stomach pains. The bark of a tree called umthombothi (Spirostachys africana) can also be used for bathing or drinking to clear rash on children …*”, explained an elderly grandmother.

## Discussion

The results of this study contribute to the basis of a deeper understanding of the dynamics of maternal nutrition, breastfeeding and IYCF practices in the context of child health, social circumstances and cultural beliefs in the Kat River Valley communities in South Africa. Both the quantitative and qualitative results have shown that participants were aware of the importance and benefits of breastfeeding but mothers rarely breastfed their children for various reasons other than HIV/AIDS. Several underlying social and behavioral factors appeared to outweigh these benefits. Formula feeding, complementary feeding and mixed feeding were the preferred infant feeding choices for the majority of mothers. Most children were weaned as early as 2 months of age, which was justified by other mothers as being due to not producing enough breast milk for their children. For those mothers who committed to breastfeeding their children, they only did so for approximately 12 months. These results are consistent with other studies in South Africa [11, 21, 23], in Zambia [7] and in Mexico [16] where formula and mixed feeding, together with complementary feeding, were the main forms of infant feeding in rural, peri-urban and urban settings. Furthermore, Fjeld et al. [17] also reported that decreased breastmilk production also forced some mothers to wean their children at an early age. Children were also given water before the age of 6 months to prevent dehydration as breastmilk alone was regarded as not having enough water. This also came as a common cultural belief in the Kat River Valley communities, that infants should be given water and herbal mixtures early in life, and mothers acquired this knowledge as advice from older relatives on how to feed and take good care of their children. These findings are also consistent with those of Fjeld et al. [7] and Mamabolo et al. [30] where water and other fluids were given to babies at an early age because of the misconception of insufficient water in breast milk. Boiled water and an herb locally known as isicakhathi (*Salvia scabra* L. f.) were given to new born babies for colic in this study and this is consistent with a study by Sibeko et al. [23] on the Xhosa mothers in Langa township, Cape Town.

The pursuit for happiness and satisfying partners was another factor that discouraged some mothers from breastfeeding their babies. Breastfeeding mothers were caught between a rock and a hard place as they were supposed to follow their culture whilst breastfeeding or risk losing their partners because of breastfeeding their children. However, both can be accommodated through formula feeding and complementary feeding, but at the expense of the child’s health. Thus, the child would not be suckling “impure” breast milk that could make them sick and at the same time, mothers would keep their partners closer. However, there was no consultation or involvement of fathers in breastfeeding decisions as mothers individually made decisions regarding weaning their children. This also highlighted the lack of involvement and support from fathers reflecting in different ways. For example, since the mothers were spending more time with their husbands or partners which is against IsiXhosa culture, either the fathers did not encourage these mothers to continue breastfeeding, or they did but their views were not taken into consideration. Ijumba et al. [11] also reported the lack of breastfeeding support from fathers in South Africa where fathers supported formula feeding by providing it for their weaned children. However, in the United Kingdom, Brown and Davies [12] reported that fathers felt left out in breastfeeding decisions and requested for specific and accessible information about the benefits of breastfeeding. Therefore, strategies to promote breastfeeding should recognize the importance of fathers, while encouraging them to support their partners to practice breastfeeding. Furthermore, the quest for freedom was also reported in others studies where formula feeding removed the barriers or lifestyle restrictions and loss of freedom associated with breastfeeding as young mothers would be able to meet with friends, boyfriends or finding new boyfriends and frequenting entertainment establishments [11, 49]. In this study, although the IsiXhosa culture is best in promoting breastfeeding, it was regarded as a barrier by young mothers as they felt it restricted them from being with their partners whilst breastfeeding.

Although the mothers in this study were familiar with the concept of infant and young child feeding and nutritious foods for child growth and development, their practices where far apart from their stated knowledge. For example, in the quantitative survey, the majority of the mothers acclaimed the importance of dairy products, vegetables and fruits in children’s diets and only 1 % mentioned niknaks as suitable food for children. However, in reality, niknaks was the second most frequently consumed food by children, whilst fruits, vegetables, eggs and flesh foods were rarely consumed. Similarly, the dietary diversity for the mothers was also very low with only 14% of women consuming a good quality diet in the previous 24 h. Both women and children were consuming diets low in essential nutrients such as grains and tubers. The diets of most women in this study were dominated by starchy staples and it was difficult for these women to provide their children with good quality diets which they failed to meet themselves. These findings are consistent with those reported by Oldewage-Theron and Kruger [50], Schönfeldt et al. [51], Faber et al. [52], Acham et al. [53] and Chakona and Shackleton [28, 37], who reported that cereals and starchy foods, dominate the diets of most poor communities in South Africa, with low intake of fruits and vegetables [28, 37, 52, 53]. Participants in Kat River Valley had some knowledge on the importance of vegetables and fruits for their children’s health, but it was impossible for them to purchase these because they are expensive and majority of families are dependent on government social grant.

Affordability was also perceived during the FDGs as one of the influencing factors for food choices and food consumption by mothers and children in the Kat River Valley communities. It is beyond the reach of many mothers to consume a more diverse diet, and let alone feeding their children with good quality, nutritious meals considering that most are unemployed and survive especially on child support grant of R410 per month. The 2018 South African Child Gauge report has shown that 79.6 and 44.9% of children in the Eastern Cape were living in income poverty households and households without an employed adult, respectively [54]. However, due to the increase in food prices, especially cereals, food consumption patterns among vulnerable households have been impacted as many have stopped to consider the quality of their food but switched to cheaper, affordable and less nutritious foods that satisfy hunger [55]. The reality is that the majority of young mothers in this study were struggling to obtain a decent income and could not afford a diverse diet, therefore they ate and fed their children with the available foods. Snacks like niknaks which are cheaper and ready to eat as well as many children’s favourite snack, made it easier for mothers to make it a meal for their children. However, niknaks were also given to children on a daily basis as a way of calming them as was reported in both quantitative and qualitative results and this has revealed the poor IYCF being practiced by mothers as was criticised by the grandmothers.

Traditional IsiXhosa foods were noted in both quantitative and qualitative results as the best food for child growth and development, as these were perceived to boost the immune system and increase children’s appetite. Furthermore, findings from this study revealed conflicts between young mothers and grandmothers, especially on what the latter deemed to be young mothers’ poor eating habits as well as poor child feeding practices which were linked to not wanting to cook or stick to traditional foods. However, all the ingredients for the mentioned traditional dishes required purchasing due to the communities’ over-dependence on food purchasing from markets. For example, foods like flour, mealie meal, samp, pumpkin, peanut butter and milk which are used to make some traditional foods are beyond the reach of many young mothers, considering that many were unemployed. This was not understood by grandmothers as they felt their traditional knowledge was going to vain. Similarly, young mothers also expressed feelings of fear regarding the preparation of the traditional foods as these could make their children sick if not well prepared. Therefore, this might have influenced their preference for modern foods, although the grandmothers felt these mothers did not want learn and cook for their children. This finding is in contrast with work in other African countries [32–34] and Nepal [31], where grandmothers’ knowledge and their decisions were regarded as critical for early child feeding practices. What was novel in this study was the ‘resistance’ to prepare traditional foods by young mothers as perceived by grandmothers, who concluded that young mothers were incapable of taking good care of their children and were therefore limited when it comes to their children’s feeding and care.

This study has both strengths and limitations. An important strength was using a mixed methodology to investigate the relationship between maternal dietary diversity, breastfeeding and IYCF practices, perceptions of mothers/caregivers and grandmothers regarding these and mothers’ eating behavior as well as the reasons for such behavior in the Kat River Valley. This makes it possible to replicate the study with other communities exhibiting the same and/or different cultures in South Africa, thus allowing a direct comparison within and between regions and cultures. Another important strength was providing stronger evidence for a conclusion through convergence and corroboration of findings from both qualitative and quantitative research which added insights and improved understanding that would have been missed when only using a single method. For example, the use of medicinal plants, the social circumstances and some cultural aspects of breastfeeding and abstinence from sexual intercourse between partners as factors causing mothers to wean their babies earlier in life which had not been mentioned during the household surveys came out during FGDs. This can however increase the generalizability of the study results because I assume that I obtained a more complete knowledge necessary to inform theory and practice. However, my study was limited to a small sample size and was conducted in five small villages under one district/municipality in the Eastern Cape province which may not be representative of the entire province and/or country. Also, some women were reluctant to talk about their food habits, dietary practices and give information about their young children, especially to someone regarded as a stranger. This may have led to an overestimation of women’s breastfeeding behavior and IYCF practices. Therefore, these results need to be interpreted with these advantages and limitations in mind.

## Conclusion

This study provides compelling evidence on how social circumstances and cultural beliefs may influence food consumption behaviour by mothers/caregivers, breastfeeding and IYCF practices in a rural South African context. Although mothers in the study were aware of the importance of breastfeeding and healthy eating, their practices did not reflect this. The lack of income, ignorance regarding preparation and use of traditional foods and exclusion of fathers in breastfeeding and child feeding decisions pose a serious concern and therefore, strategies should incorporate paternal inclusion in breastfeeding decisions and advocate for the promotion to safeguard and use indigenous knowledge in IYCF practices by young mothers. Furthermore, the importance of striking a balance between mothers’ income, cultural beliefs, breastfeeding and life of lactating mothers so that they will not feel burdened and isolated when taking care of their little children is required. However, there is need for further research to examine the importance of fathers’ involvement, their support to their breastfeeding partners as well as their opinion regarding breastfeeding, in the South African context.

## Data Availability

The datasets used and/or analysed during the current study are available from the corresponding author on reasonable request.

## Footnotes

1 Complementary feeding refers to introduction of solid foods in infant’s diet

2 Weaning refers to removal of breast milk from infant’s diet

3 Matric qualification refers to the final year of high school and the qualification received on graduating from high school which gives a person the minimum university entrance requirement.

4 A South African food consisting of dried corn kernels coarsely crushed but not as fine as mealie-meal or mealie-rice.
